# Ulinastatin Protects against Acute Kidney Injury in Infant Piglets Model Undergoing Surgery on Hypothermic Low-Flow Cardiopulmonary Bypass

**DOI:** 10.1371/journal.pone.0144516

**Published:** 2015-12-14

**Authors:** Xiaocou Wang, Qinghua Xue, Fuxia Yan, Jinping Liu, Shoujun Li, Shengshou Hu

**Affiliations:** 1 Department of Anesthesiology, Critical Care and Pain Medicine, the Second Affiliated Hospital, Wenzhou Medical University, Wenzhou, Zhejiang, China; 2 Department of Anesthesiology, State Key Laboratory of Cardiovascular Disease, Fuwai Hospital, National Center for Cardiovascular Diseases, Chinese Academy of Medical Sciences and Peking Union Medical College, Beijing, China; 3 Department of Cardiopulmonary Bypass, State Key Laboratory of Cardiovascular Disease, Fuwai Hospital, National Center for Cardiovascular Diseases, Chinese Academy of Medical Sciences and Peking Union Medical College, Beijing, China; 4 Department of Surgery, State Key Laboratory of Cardiovascular Disease, Fuwai Hospital, National Center for Cardiovascular Diseases, Chinese Academy of Medical Sciences and Peking Union Medical College, Beijing, China; Imperial College London, Chelsea & Westminster Hospital, UNITED KINGDOM

## Abstract

**Objective:**

Infants are more vulnerable to kidney injuries induced by inflammatory response syndrome and ischemia-reperfusion injury following cardiopulmonary bypass especially with prolonged hypothermic low-flow (HLF). This study aims to evaluate the protective role of ulinastatin, an anti-inflammatory agent, against acute kidney injuries in infant piglets model undergoing surgery on HLF cardiopulmonary bypass.

**Methods:**

Eighteen general-type infant piglets were randomly separated into the ulinastatin group (Group U, n = 6), the control group (Group C, n = 6), and the sham operation group (Group S, n = 6), and anaesthetized. The groups U and C received following experimental procedure: median thoracotomy, routine CPB and HLF, and finally weaned from CPB. The group S only underwent sham median thoracotomy. Ulinastatin at a dose of 5,000 units/kg body weight and a certain volume of saline were administrated to animals of the groups U and C at the beginning of CPB and at aortic declamping, respectively. Venous blood samples were collected at 3 different time points: after anesthesia induction in all experimental groups, 5 minutes, and 120 minutes after CPB in the Groups U and C. Markers for inflammation and acute kidney injury were tested in the collected plasma. N-acetyl-β-D-glucosaminidase (NAG) from urine, markers of oxidative stress injury and TUNEL-positive cells in kidney tissues were also detected.

**Results:**

The expressions of plasma inflammatory markers and acute kidney injury markers increased both in Group U and Group C at 5 min and 120 min after CPB. Also, numbers of TUNEL-positive cells and oxidative stress markers in kidney rose in both groups. At the time point of 120-min after CPB, compared with the Group C, some plasma inflammatory and acute kidney injury markers as well as TUNEL-positive cells and oxidative stress markers in kidney were significantly reduced in the Group U. Histologic analyses showed that HLF promoted acute tubular necrosis and dilatation.

**Conclusions:**

HLF cardiopulmonary bypass surgery could intensify systemic inflammatory responses and oxidative stress on infant piglets, thus causing acute kidney injury. Ulinastatin might reduce such inflammatory response and oxidative stress and the extent of kidney injury.

## Introduction

In the last decades, improved perfusion techniques and perioperative interventions, corrected congenital heart defects at a low operative mortality in more infant and children patients at an early stage [1]. However, a wide range of postoperative end-organ complications including brain, renal, pulmonary, and myocardial injuries remains after operations [2,3]. Acute kidney injury(AKI) is one of the major complications of cardiac patients undergoing surgery on cardiopulmonary bypass (CPB)[4]. Drs. Kumar [5] and Mariscalco [6] report the incidence of acute kidney injury is as high as 6.6% to 40% after CPB, and that 1% to 6% of such patients need long-term dialysis treatments.

Hypothermic low-flow (HLF) cardiopulmonary bypass, a noval perfusion technique, provides clear vision, good handling conditions, and a high surgical success rate during the operation. HLF technique has been frequently used for complex congenital heart defects surgeries in patients at all ages, especially in neonates and infants. With undeveloped renal regulatory function, the patients are more susceptible to acute kidney injuries after CPB.

Recently, Li and colleagues [7] report that ulinastatin could be an effective treatment for AKI following liver transplantation in rats and humans and their study suggests that ulinastatincan protect against AKI following orthotopic liver transplantation by inhibiting inflammation and oxidation. However, the effectiveness of ulinastatin in the treatment of AKI caused by HLF and its mechanisms are still unknown. This study was designed to determine the effectiveness of ulinastatin on alleviating HLF induced AKI in infant piglet models.

## Materials and Methods

### Animal care and study protocol

General-type infant piglets (14 to 18 days old, weighting 3.0 to 6.2 kg) were supplied by Beijing Huaige Farms. All protocols in this study were approved by the Committee on the Ethics of Animal Experiments of Fuwai Hospital, Peking Union Medical College and the Beijing Council on Animal Care, Beijing, China (IACUC permit number: FW2010-101523), in compliance with the Guide for the Care and Use of Laboratory Animals published by the US National Institutes of Health (NIH publication no.85-23, revised 1996). All reasonable efforts were made to minimize animal suffering and to use only the number of animals necessary to produce reliable scientific data. Eighteen infant piglets were assigned into the ulinastatin group (Group U, n = 6), the control group (Group C, n = 6), and the sham operation group (Group S, n = 6) by computer-generated randomization. Ulinastatin at 2,500 IU/kg (Ulinastatin for Injection; Techpool Bio-Pharma Co., Ltd, Guangdong, China) was defined as a bolus infusion, respectively when CPB was initiated and when the aortic cross-clamp was removed in the Group U (totally received 5,000 IU/kg) [8, 9]. Equivalent volumes of normal saline was administered in the Group C at the same times. Animals in the Group S were not accepted CPB and only were performed sternotomy and 360 minutes general anesthesia.

### Anesthesia and monitoring

Procedures in the pediatric cardiac perioperative management and anesthesia was performed in order to maximize survival rate and experimental reliability, as detailed in our previous study [10]. Briefly, animals were respectively fasted formula for 6 hours and water for 2 hours before anesthesia. During basic anesthesia, ketamine (10mg/kg), midazolam (0.5mg/kg), and atropine (0.05mg/kg) were administered intramuscularly. For each animal, an ear vein access was established and a urine catheter was inserted. Animal were connected to a monitor (the IntelliVue MP60; Philips Medizinsysteme, Boeblingen, Germany), to monitor nasopharyngeal temperature (NT); electrocardiogram (ECG) with electrodes pasted on the distal limbs following skin preparation; pulse oximetry with an ear clip-oximetry probe; invasive arterial blood pressure (ABP) with 20G trocar catheterized in right carotid artery after cervical partial incision; central venous pressure (CVP), with 5Fr double-lumen central venous catheter catheterized in right external jugular vein after the incision. The cervical partial incision was performed at the beginning of maintenance anesthesia phase just after induction of anesthesia. Induction and maintenance of anesthesia were performed as following steps. Fentanyl citrate (20μg/kg), pipecuronium bromide (1.5mg/kg) and midazolam (0.25mg/kg) was administered as a bolus to each animal through ear veins, then manual breathing was performed using asimple breathing mask, and finally a cuffed endotracheal tube with internal diameter 4.0mm was intubated after the glottis exposed with anesthesia laryngoscope and was connected to an anesthetic machine (Dräger Primus; Dräger Co, Lübeck, Germany) with its breathing pattern in the volume control mode. Initial ventilatory parameters were setup as inspired oxygen fraction at 1.0; respiratory rate at 30 per minute; delivered tidal volume at 10 ml/kg; positive end-expiratory pressure (PEEP) at 4 cm H_2_O; endtidal carbon dioxide tension ranging from 35 to 40 mmHg; and inspiratory timeat 0.65 s. Anesthesia was maintained by administering intermittently boluses of narcotics and the drugs used in anesthesia induction at the following time points: skin incision, median thoracotomy, cardiopulmonary bypass. Experimental animals were given 1% sevoflurane by an anesthesia machine or a CPB circuit when vena cava were blocked and CPB started. Appropriate treatments were used to maintain stable hemodynamics and internal environment based on monitoring data and arterial blood gas analysis. Following indexes of blood gas analysis were tried to maintained: pH: 7.35~7.45; oxygen partial pressure: 80~120mmHg; carbon dioxide partial pressure: 35 ~ 45mmHg; oxygen saturation: 95%~100%; residual alkali: -2.3~2.3mmol/L; hemoglobin: >85g/L; sodium ions: 135 ~ 145mmol/L; and potassium ion: 3.5~4.5mmol/L.

### Surgical procedure, cardiopulmonary bypass and HLF

Following median sternotomy and heparinization with heparin at 400 IU/kg, a conventional nonpulsatile systemic CPB flow was established using an 8 Fr aortic cannula in the ascending aorta and a 22 Fr venous cannula in the right atrial appendage. Sterile CPB circuits (Tianjin Plastics Research Institute, Tianjin, China) primed with 400 ml donor porcine blood, 200 ml hydroxyethyl starch (6%), 5ml sodium bicarbonate solution (5%), and heparin (400 IU/kg) bridging cannulas with roller pumps (Jostra HL20; Maquet Co, Solna, Sweden), membrane oxygenator (Capiox; Terumo Co, Tokyo, Japan), and standard arterial filter (Xijing Co, Xian, China). When NT was decreased to 30°C through a moderate systemic hypothermia (Jostra Heater Cooler Units, Maquet Co, Solna, Sweden), cardiac arrest was obtained by aortic cross-clamp and antegrade infusion of St. Thomas’ cardioplegic solution at 4°C at an initial dose of 20 ml/kg and a maintain dose of 10 ml/kg every 30 min via a cannula inserted in the aortic root. Iced saline was placed in the pericardium for further myocardial protection. Left atrial drainage was established and the ventilator was in standby mode during the cardiac arrest period. When body temperatures of animals were gradually decreased to 25°C, arterial pump flow down-regulated to 50 ml/kg/min (HLF). After 120 min of cardiac arrest, NTs were up-regulated to 30–32°C, aortic cross-clamps were removed and dopamine was infused at 5μg/kg/min and adjusted by hemodynamic indexes. In the next 30 min, animals underwent assisted circulation and modified ultrafiltration, were rewarmed to 35–36°C, and finally were weaned from CPB. Ventilations as mentioned above were given when cardiac resuscitation. In the meanwhile, protamines (1.3 mg *vs* 100 IU heparins) were given to reverse heparinization and finally sternums were closed. Then, animals were observed for 120 min and sacrificed by intravenous hyperkalemic injection. Piglets failed to wean from CPB were excluded and replaced in this study.

### Measurements

#### Measurements of the blood and urine samples

Venous blood samples collected immediately after anesthesia induction (T1) as a baseline, at the 5 min (T2) and 120 min (T3) weaned from CPB in Group U and Group C. All samples were handled and transported using iceboxes and centrifuged at the speed of 3,000xg for 15 minutes at 5°C. Urine samples were obtained via urine catheters at the end of each experiment. Supernatants were stored at -80°C for subsequent chemical analysis.

Concentrations of kidney injury marker, cystatin C (CysC) and plasma inflammatory factors (interleukin-6 (IL-6), tumor necrosis factor-α (TNF-α)) were detected using porcine enzyme-linked immunosorbent assays (ELISA) kits (R&D Systems, Inc., Minneapolis, MN, USA). Serum creatinine (SCr), a kidney injury marker, and blood urea nitrogen (BUN) were measured with an automatic biochemical analyzer (UniCel DXC800 Synchron, Beckman Coulter Inc, CA, USA). Urine N-acetyl-β-D-glucosaminidase (NAG) was tested by particles-enhanced nephelometric immunoassays (Dade Behring Inc, CA, USA). All experiments were performed according to manufacturer’s instructions.

#### Measurements of the kidney samples

Tissue samples of the upper segment of right kidneys were collected immediately after the animal demise and were divided into two groups. One group of tissue samples would be frozen in liquid-nitroge and further used to examine oxidative stress markers, superoxide dismutase (SOD) and malondialdehyde (MDA) with ELISA kits (R&D Systems, Inc., Minneapolis, MN, USA). The other group of tissue samples was utilized for tissue pathology test. Experimental procedures were specified as follows, samples were rinsed, cleared of connective tissues with 10×PBS buffer at 4°C for 48 hours, then fixed in 10% formalin, embedded in paraffin, cut into 5 μm thick sections, and finally stained with hematoxylin-eosin (H&E), deoxyuride-5’ -triphosphate biotin nick end labeling (TUNEL), an apoptotic detection (Roche Diagnostics, Mannheim, Germany). Fifty four photographs from H&E stained slides (three slides from each animal) were examined by two independent pathologists blinded to the experiments, and evaluated the degree of renal damage (tubular cell necrosis, cytoplasmic vacuole formation, hemorrhage, and tubular dilatation) using a semi-quantitative score method [A high score represents more severe damage: maximum score is 4; normal kidney is 0; minimal damage is (0–5% involvement); mild damage is 2 (5–25% involvement); moderate damage is 3 (25–75% involvement); and severe damage is 4 (75–100% involvement)][4] using a light microscopy (Olympus Corp, Tokyo, Japan). Fifty-four images from TUNEL tests were examined. TUNEL-positive renal cells as well as the number of total cells were counted with Leica Qwin plus V3 software (Nussloch, Germany) and the percentage of TUNEL positive renal cells to the total cells was employed for statistical analyses.

### Power of the study and statistical analysis

Increase in SCr to ≥1.5 times baseline, which is known or presumed to have occurred within the prior 7 days, as an index of definitions of acute kidney injury[11], was the primary endpoint, and assuming SCr to <1.5 times baseline in ulinastatin group.With preliminary experiments (2 animals in each group), 2h after CPB, SCr levels were increased(86.5±12.02μmmol/L in control group vs 73.2±16.9μmmol/L, with 51.4±9.8 vs 52.6±14.6 as baseline). Therefore, we calculated that a study with 12 animals (six per group) would have a 90% power to detect a large effect size of 0.7 standard deviations, equivalent to a difference of 21.8μmmol/L in SCr between groups assuming a within group standard deviation of 13.5. Extra animal experiments (n = 6) were added as sham operation group to excluding the impact of median sternotomy on renal function. All data were expressed as mean ± standard deviation. Plasma/urine markers of inflammation, oxidative stress and kidney injury were compared between groups by the single factor analysis of variance. The LSD-t test was used to perform pairwise comparisons. Data at different time points were analyzed by variance analysis of repeated measures. Data for nonrepetitive measurements were analyzed by one-way analysis of variance (ANOVA) for comparisons between any two groups. All data were analyzed with a commercially available statistical software package (SPSS for Windows version 13.0; SPSS Inc, Chicago, IL, USA) and statistically significance was setup at *P* < 0.05.

## Results

### Perioperative general parameters

No animal in the three groups was excluded in the experiments. Perioperative general parameters are presented in Table 1. No significant differences were found in age, weight, gender, durations of anesthesia, duration of CPB, and duration of aortic clamped among three groups (*P* >0.05).

**Table 1:** Perioperative general parameters of the three groups.

	Group C (n = 6)	Group U (n = 6)	Group S (n = 6)
Age (days)	16.50±1.87	16.83±3.06	19.00±2.3
Weight (kg)	5.21±0.52	5.47±0.48	5.83±0.42
Gender	4/6 Female	4/6 Female	3/6 Female
Duration of anesthesia (min)	341.64±17.33	363.69±26.42	359.50±5.86
Duration of CPB (min)	194.73±14.48	187.84±11.69	--
Duration of aortic clamped (min)	129.28±15.38	137.92±14.75	--

Data are shown as mean ± SD.

### Plasma markers of inflammation

**Fig 1:**
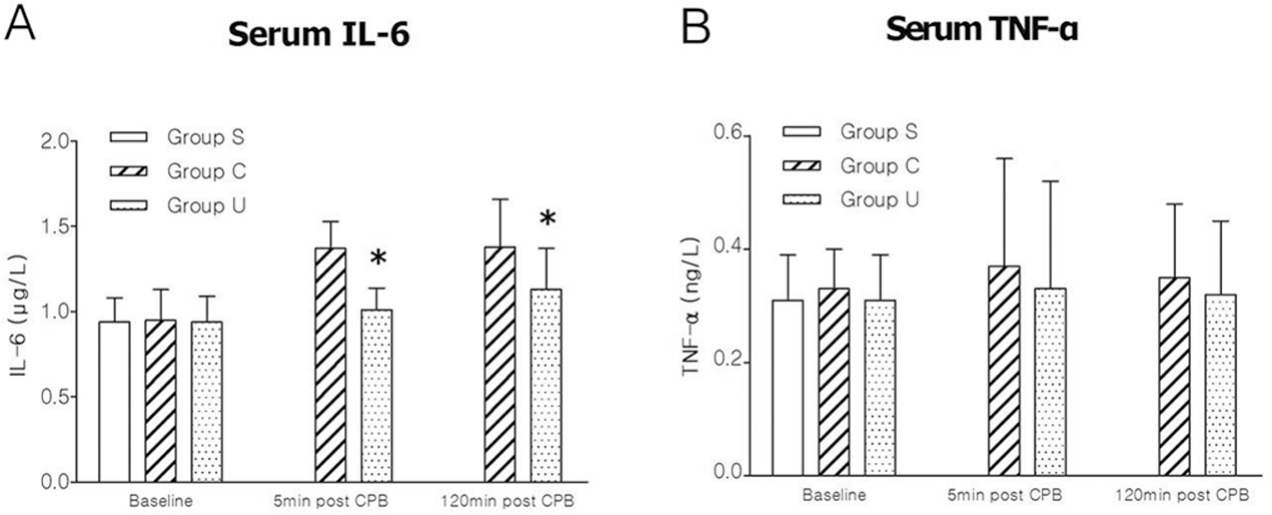
Results of plasma markers of inflammation in the three groups. (A) serum IL-6, interleukin-6; (B) serum TNF-α, tumor necrosis factor-α. Data are presented as mean±SD, n = 6, *P <0.05 versus Group C. For graphs pooled estimates for pairwise comparisons derived from Analysis of Covariance with adjustment for baseline serum IL-6 at 0.94±0.13μg/L, serum TNF-α 0.32±0.05ng/L, were as follows: **IL-6;** 5min post CPB(T2): Group C, 1.37±0.16μg/L, Group U, 1.01±0.13μg/L. Test for overall treatment effect *p* = 0.021. 120min post CPB(T3): Group C, 1.38±0.28μg/L, Group U, 1.13±0.24μg/L. Test for overall treatment effect *P* = 0.001. **TNF-α**; 5min post CPB(T2): Group C, 0.37±0.19 ng/L, Group U, 0.33±0.19ng/L. Test for overall treatment effect *P* = 0.075. 120min post CPB(T3): Group C, 0.35±0.13 ng/L, Group U, 0.32±0.13 ng/L. Test for overall treatment effect *P* = 0.088.

Data of plasma markers of inflammation are presented in Fig 1. For IL-6 (Fig 1A) and TNF-α (Fig 1B), no statistical significance was found at T1 among three groups (all *P* >0.05), but an increasing trend after CPB both in Group C and Group U. At T2, IL-6 and TNF-α decrease by 26.28% and 10.81% in Group U respectively compared to Group C. At T3, IL-6 and TNF-α declined by 18.12% and 8.57% respectively in Group U, compared to Group C.

### Markers of oxidative stress injury in kidney

Data of oxidative stress injury markers MDA and SOD in kidney are presented in Table 2. MDA of kidney tissues in the Group S was very low and significantly increased after HLF in the Group C, while exposure to ulinastatin in the Group U could inhibit the increase. The variation of SOD was in a contrary situation in the three groups.

**Table 2:** MDA and SOD in kidney tissues in three groups.

	Group S (n = 6)	Group C (n = 6)	Group U (n = 6)
MDA (nmol/μL)	10.07±2.33	24.62±3.57^a^	15.57±2.23 ^a^ ^,^ ^b^
SOD (U/μL)	153.74±17.84	83.58±9.63 ^a^	99.85±10.75 ^a^ ^,^ ^b^

Data are shown as mean ± SD, compared with the Group S

^a^ *P* <0.05, compared to the Group C

^b^ *P* <0.05.

### Markers of kidney injury

**Fig 2:**
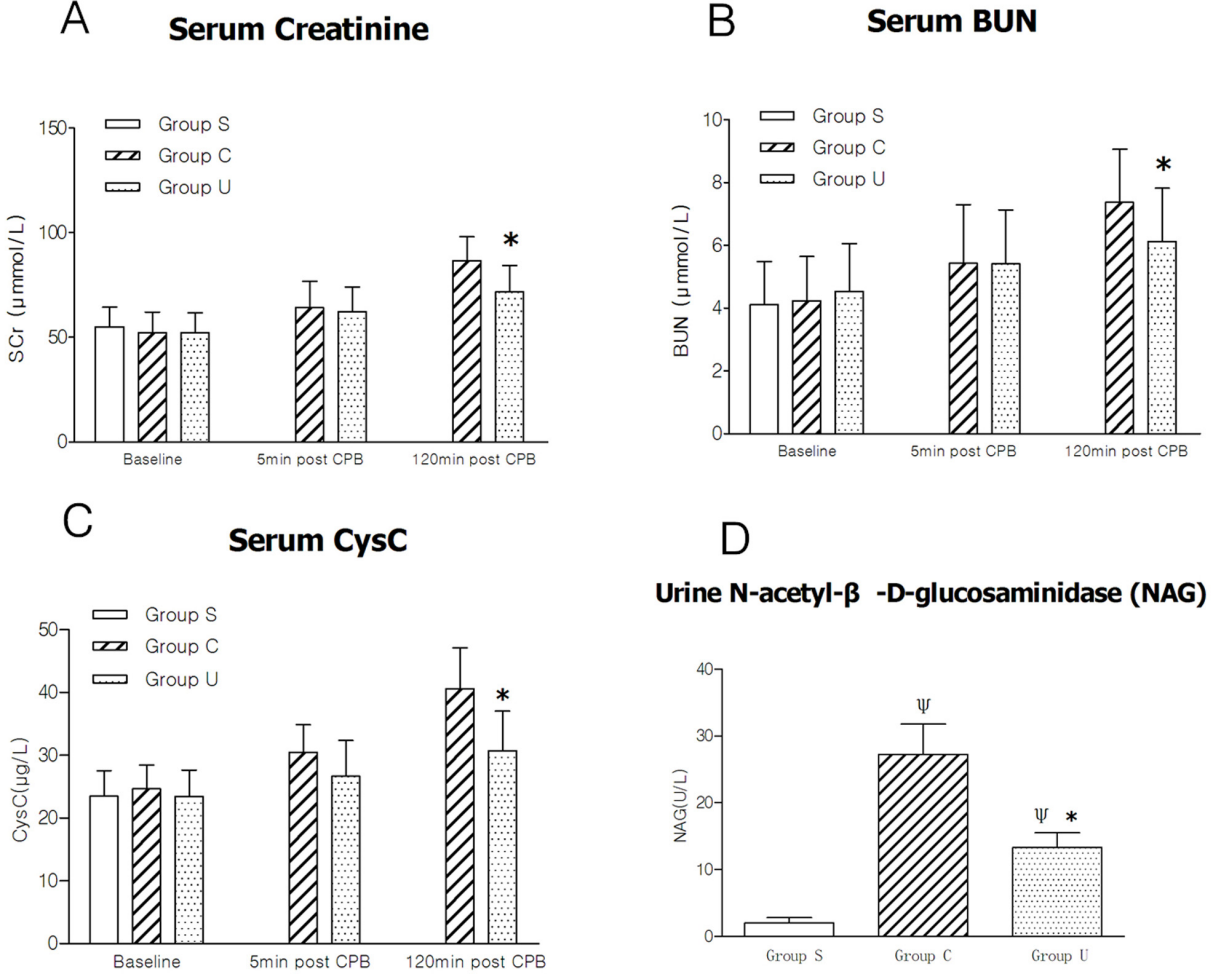
Results of markers of kidney injury in the three groups. (A) serum creatinine; (B) serum BUN; (C) serum CysC; (D) urine NAG, urine N-acetyl-β-D-glucosaminidase. Data are presented as mean±SD, n = 6, *P <0.05 versus Group C, Ψ*P* <0.05 versus Group S. For graphs pooled estimates for pairwise comparisons derived from Analysis of Covariance with adjustment for baseline serum creatinine at 53.61±9.53μmmol/L, serum BUN 4.31±1.34μmmol/L, serum CysC 24.35±4.2μg/L, were as follows: **serum creatinine;** 5min post CPB(T2): Group C, 64.24±12.53μmmol/L, Group U, 62.33±11.73μmmol/L. Test for overall treatment effect *P* = 0.074. 120min post CPB(T3): Group C, 86.62±11.41μmmol/L, Group U, 71.72±12.55μmmol/L. Test for overall treatment effect *P* = 0.032. **serum BUN;** 5min post CPB(T2): Group C, 5.43±1.87μmmol/L, Group U, 5.41±1.72μmmol/L. Test for overall treatment effect *P* = 0.081. 120min post CPB(T3): Group C, 7.37±1.72μmmol/L, Group U, 6.13±1.69μmmol/L. Test for overall treatment effect *P* = 0.025. **serum CysC;** 5min post CPB(T2): Group C, 30.47±4.4μg/L, Group U, 26.66±5.7μg/L. Test for overall treatment effect *P* = 0.069. 120min post CPB(T3): Group C, 40.62±6.5μg/L, Group U, 30.72±6.2μg/L. Test for overall treatment effect *P* = 0.001.

At T1, plasma concentrations of SCr (Fig 2A), BUN (Fig 2B), and CysC (Fig 2C) were very low, and no statistical significance was found among the three groups (*P* >0.05). These three markers also tended to increase after CPB both in the Groups C and U. At T2, SCr, BUN, and CysC respectively reduced by 2.97%, 0.37% and 12.50% in the Group U, compared to the Group C. At T3, compared to the Group C, SCr, BUN, and CysC, decreased respectively by 17.20%, 16.82%, and 24.37%, in the Group U. For urine NAG (Fig 2D), statistical significance was found between the Group C and the Group U (13.30±2.23 U/L in Group U *vs* 27.24±4.53 U/L in Group C, *P* = 0.02). All data are presented as Fig 2.

### Histologic examination and TUNEL assays

**Fig 3:**
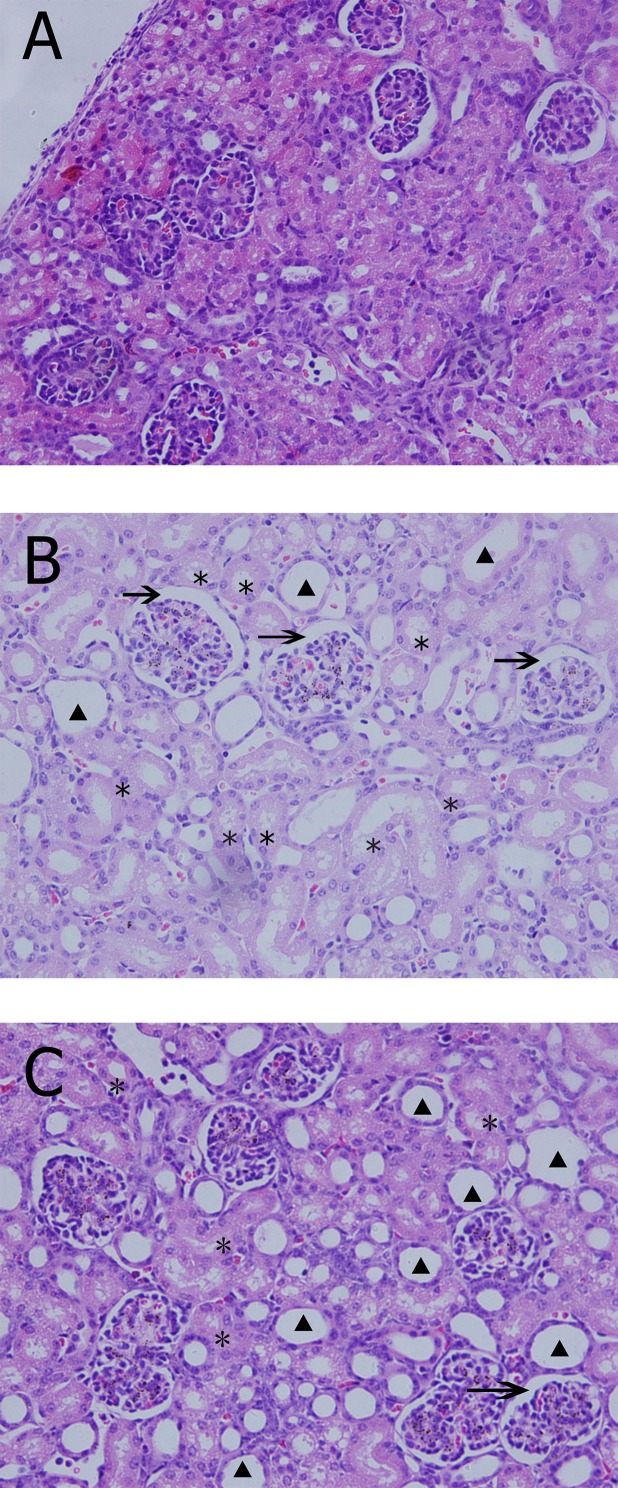
Typical histological examination results in the three groups. A, Group S, Tubules and glomeruli appear normal (H&E×400); B, Group C, after 2h CPB, kidney histologic changes include tubular dilatation(*), vacuole formation(▲), and glomerular over-filling (arrow) appeared obvious (H&E×400); C, Group U, injury changes of kidney still exist as tubular dilatation(*), vacuole formation(▲), and glomerular over-filling (*arrow*) but were milder with intervention of ulinastatin(H&E×400).

The Group S exhibited normal structure of kidney tissues (Fig 3A). Histologic changes, including tubular dilatation, tubular necrosis, vacuole formation, and glomerular over-filling were observed in the Group C (Fig 3B). In the Group U, the structure of kidney tissues was not clear, and the injury presented to a lesser extent (Fig 3C). The results from semi-quantitative scoring analyses also supports the finding. Pathologic damages scores correlated significantly with the Group C and the Group U (3.21 ± 0.5 in Group C vs 1.80 ± 0.4 in Group U, *P* = 0.02), compared to the Group S (0.33 ± 0.52), CPB groups showed statistically significance (both *P* < 0.05).

The percentage of TUNEL-positive cells both in the Groups C and U were significantly more than that in the Group S (7.87 ± 0.69/1000 vs. 2.58 ± 0.36/1000, *P* = 0.000 and 4.87 ± 0.78/1000 vs. 2.58 ± 0.36/1000, *P* = 0.003). However, the percentage of TUNEL-positive cells decreased in the the Group U compared to that in the Group C (7.87 ± 0.69/1000 vs. 4.87 ± 0.78/1000, *P* = 0.005).

## Discussion

Due to the complexity in establishing CPB, common animal models are adult dogs [12], pigs [2, 13, 14] and other large animals, whereas infant animals are rarely used in relevant experiments. In a study using a big animal model, significant differences of perioperative management in clinical practice appear. The main defects of the study include a long time water fasting, tracheal intubation without muscle relaxant, failure of timely adjusting internal environments based on results of blood gas analyses. For an infant animal model, it has limited renal functions and could be easily avoid the two experimental flaws mentioned above. When moderate or severe water shortages, and internal environmental disorders cannot be corrected timely during surgery, renal functions would be impaired. In this study, management procedures of clinical pediatric cardiac perioperative including people (a dedicated team of anesthesiologists, surgeons, perfusionists, and nurses), equipments, drugs and supplies were employed, leading to a high successful rate using infant piglet CPB model. Also, no animal was excluded or replaced in the study.

HLF bypass strategy is frequently used in complex congenital heart defects surgeries for infants and children and HLF should be prolonged based on the complexity of defects. In this study, after 2 hour HLF, acute kidney injury can be happened not only with the increase of markers as CysC, SCr, BUN in plasma and NAG in urine but also with pathologic damage and increased apoptosis rate in kidney tissue in the Group C. This indicates a certain kidney injury accompanied with prolonged HLF in infant piglets, which is consistent with the majority of similar studies in the literature [14–16]. Among kidney injury markers, SCr and BUN are the most widely used markers in the last 40 years. CysC is an endogenous cysteine proteinase inhibitor and is produced by nucleated cells at a constant rate. It is freely filtered, reabsorbed, and catabolized by the glomeruli, but it is not secreted by the tubules. CysC is a useful detection marker in acute kidney injury reflecting disease severity, prognosis superior to other markers such as serum creatinine and blood urea nitrogen [17,18]. NAG is a lysosome hydrolase widely distributing in various tissues and cells with a molecular weight of approximately 140 kDa which limits NAG molecules to be filtered through the glomeruli. Urine NAG is a novel indicator more sensitive than SCr which can be used to evaluate an early damage to epithelial cells in proximal convoluted tubules during the progression of renal diseases and is also an index of renal tubular damage [19].

Postoperative acute kideny injury is thought to be the consequence of an interplay of different pathophysiologic mechanisms. Systemic inflammatory responses, ischemia-reperfusion and oxidative stress injuries in kidney induced by CPB, and so as the peculiar blood circulation of kidney, all those factors are considered as relevant determinants of postoperative AKI[6]. During CPB, surgical trauma, organs ischemia-reperfusion and CPB relevant monocyte-macrophages, neutrophils and other inflammatory cells can be recruited and activated, release of inflammatory cytokines can be induced by blood components contacting with the surfaces of the artificial heart-lung machine. Inflammation-mediated cytokines interact with inflammatory cells and finally induce inflammatory cascade by amplification reactions trigger systemic inflammatory response syndrome (SIRS) eventually leading to renal damages [6]. Additionally, tubular obstruction caused by free hemoglobin results in renal tubular cell necrosis, which exacerbates the renal injury [20]. Interleukin-6 (IL-6) and tumor necrosis factor-alpha (TNF-α) are plasma markers of early inflammation response initiating the cascade of activation of other cytokines in the inflammatory response and final attributing to SIRS [4]. The increasing extent of IL-6 and TNF-α can reflect serious degrees of kidney injury. In this study, plasma IL-6 and TNF-α increased critically both at 5-min and 120-min after the weaning from CPB, indicating that prolonged HLF cardiac surgery could cause a significant inflammatory reaction. Ischemia-reperfusion and oxidative stress injury is another mechanism of kidney injury undergoing CPB in combination with systemic inflammatory responses. Target organ's concentrations of MDA and SOD were commonly examined to evaluate the severity of oxidative stress injury [12]. MDA is an end-product of lipid peroxidation and also an indicator of the extent of lipid peroxidation as well as the degree of tissue injury induced by oxygen free radicals. SOD is an oxygen free radical scavenger reflecting the ability of scavenging oxygen free radicals. In this study, MDA in kidney increased and SOD decreased significantly after HLF, demonstrating that kidney is one of the severe target organs oxidative stress injured by prolonged HLF.

Ulinastatin is a broad-spectrum hydrolase inhibitor (a molecular weight of 67 000 daltons) and is purified from fresh urine of healthy men. It can have anti-inflammatory effects by inhibiting various inflammatory proteases such as trypsin, chymotrypsin, and neutrophil elastase, and plasmin [21]. Considering its effective anti-inflammatory effects, and ulinastatin has been widely used for pancreatitis, rheumatoid arthritis, sepsis, and other inflammatory diseases in China, Korea, and Japan [21–23]. This study shows that renal injury indicators CysC, SCr, and BUN in the Group U were lower than those in the Group C, 5 and 120 min weaning from CPB under the exposure to ulinastatin. Pathological examination also found kidney injury in the experimental groups was milder than the control group. The results indicate ulinastatin could protect against acute kidney injury in infant piglets undergoing HLF. Plasma IL-6, TNF-α and MDA of kidney tissues in the Group U were less than those in the Group C while SOD in the Group U is more than that in the Group C. The finding indicates ulinastatin can reduce inflammation by inhibiting the release of a variety of inflammatory cytokines, and relieve oxygen free radical damage caused by ischemia and reperfusion. Furthermore, Nishiyama and colleague's [24] studies suggest ulinastatin can prevent red blood cells from destructing and reduce free hemoglobin in blood, resulting in free hemoglobin obstructing renal tubular.

In this study, we found that prolonged HLF could cause actue renal injury, ulinastatin dose-dependently ameliorated CPB-induced kidney injury, probably mediated by inhibiting inflammatory response and oxidative stress.

## Limitations

Several limitations exist in this study. Above all, only two indicators of early inflammation (IL-6 and TNF-α) were tested, upstream factors (eg the related mRNA) and related regulatory factors (such as NF-κB) were not detected in our study. Therefore, the target link in the inflammation chain which ulinastatin inhibited and its mechanism remains to further explore. Then, infant piglets were sacrificed 120min after weaning CPB, long-term effects on renal functions were not investigated. Additionally, this study was not focused renal vascular permeability. CPB-induced acute inflammation and renal ischemia-reperfusion injury can increase renal vascular permeability. It is necessary to further test whether ulinastatin's protective effects on kidney are mediated via increasing renal vascular permeability.
